# Importance of Landraces in Cereal Breeding for Stress Tolerance

**DOI:** 10.3390/plants10071267

**Published:** 2021-06-22

**Authors:** Daniela Marone, Maria A. Russo, Antonia Mores, Donatella B. M. Ficco, Giovanni Laidò, Anna M. Mastrangelo, Grazia M. Borrelli

**Affiliations:** Council for Agricultural Research and Economics, Research Centre for Cereal and Industrial Crops, S.S. 673, Km 25,200, 71122 Foggia, Italy; daniela.marone@crea.gov.it (D.M.); mariaanna.russo@crea.gov.it (M.A.R.); antonia.mores@crea.gov.it (A.M.); donatellabm.ficco@crea.gov.it (D.B.M.F.); giovanni.laido@crea.gov.it (G.L.); annamaria.mastrangelo@crea.gov.it (A.M.M.)

**Keywords:** landraces, cereal crops, genetic resource conservation, abiotic and biotic stresses, breeding

## Abstract

The renewed focus on cereal landraces is a response to some negative consequences of modern agriculture and conventional breeding which led to a reduction of genetic diversity. Cereal landraces are still cultivated on marginal lands due to their adaptability to unfavourable conditions, constituting an important source of genetic diversity usable in modern plant breeding to improve the adaptation to abiotic or biotic stresses, yield performance and quality traits in limiting environments. Traditional agricultural production systems have played an important role in the evolution and conservation of wide variability in gene pools within species. Today, on-farm and ex situ conservation in gene bank collections, together with data sharing among researchers and breeders, will greatly benefit cereal improvement. Many efforts are usually made to collect, organize and phenotypically and genotypically analyse cereal landrace collections, which also utilize genomic approaches. Their use in breeding programs based on genomic selection, and the discovery of beneficial untapped QTL/genes/alleles which could be introgressed into modern varieties by MAS, pyramiding or biotechnological tools, increase the potential for their better deployment and exploitation in breeding for a more sustainable agricultural production, particularly enhancing adaptation and productivity in stress-prone environments to cope with current climate changes.

## 1. Introduction

The growing increase of human population and environmental stresses have led to land use changes and habitat destruction in order to produce a greater amount of food. The advent of high input and intensive monoculture systems, based on genetically similar individuals (elite cultivars, pure lines, hybrids, or clones) improved for particular traits (i.e., high yield and better end-use quality) and derived from a relatively narrow germplasm pool, resulted in a loss of potentially useful traits, reducing genetic variability that can be used by plant breeders for crop improvement. To cope with climate change and meet the needs of new varieties for marginal areas, researchers and breeders are constantly looking for new sources of genetic variability. A key component of agro-biodiversity is represented by landraces.

Landraces adapt to specific agro-climatic conditions while maintaining considerable diversity between and within populations [1], constituting a reservoir of genetic diversity that is interesting for future breeding work as well as for the development of new agricultural systems and new products. Therefore, the exploration of their genetic diversity and conservation for future generations is important.

Landraces have been described by Harlan [2] as populations that had evolved in subsistence agricultural societies as a result of “millennia-long”, “artificial” human selection pressures, mediated by human migration, seed exchange and natural selection. The term has been extended, considering the association with marginal environments and the lack of direct competition with highly bred cultivars [3]. Moreover, the definition, which well describes landrace complexity, is that “it is a dynamic population(s) of a cultivated plant that has historical origin, distinct identity and lacks formal crop improvement, as well as often being genetically diverse, locally adapted and associated with traditional farming systems” [4,5]. More generally, landraces have been proposed as autochthonous when cultivated for more than a century in a specific region, or allochthonous if introduced in another region due to migration (known as seed flow) [6]. A third type, called ‘Creole’ landrace, is a bred variety in origin, which will become landrace after numerous repeated cycles of sowing and selection by the farmer in a specific place [7,8].

Cereal landraces emerged in different regions of the world as a result of centuries of crop evolution in traditional agrosystems, leading to heterogeneous populations rather than a few superior genotypes [9,10]. Farmers have been the keepers of cereal diversity [11], selecting genetic materials with desired traits and saving seeds for subsequent growing seasons [12] and increasing gene flow through seed exchanges with relatives and neighbours or through the introduction of local or exotic materials. The exchange of seeds between farmers ensures the maintenance of the genetic heterogeneity of the landraces, which can contribute to the creation of new local varieties (populations), as well as groups of interrelated local varieties (which could be considered as meta-populations) [13].

Over time, the ageing of rural populations and mass migration of young workers from rural areas to cities have resulted in the abandonment of traditional agricultural practices and have created serious threats for the cultivation and possible use of landraces as a source of biodiversity [11,14]. The situation is further aggravated by the scarcity of national inventories of landraces and, more generally, by the absence of national institutions responsible for their conservation [15]. Despite this, cereal landraces are still cultivated on marginal lands due to their adaptability to unfavourable conditions [16]. In selfing species, the genetic diversity, held together as gene blocks in low-frequency recombination chromosomal regions, can confer a specific adaptation to stress environments [10]. Thus, landraces provide an interesting model for mapping genes that control adaptive variation in crop species [17,18,19,20].

This review presents an overview of the different aspects related to the conservation of the main cereal landraces and how these collections can be useful for modern breeding. Landrace collections have been extensively characterized in terms of genetic diversity and population structure and have a huge potential for the identification of genetic factors that are valuable for improving the important agronomic traits of cereal crops such as resistance to biotic and/or abiotic stresses, thus sustaining grain yield and quality in unfavourable environments.

## 2. Conservation ‘In Situ’ and ‘Ex Situ’ of the Main Cereal Collections and Availability of Databases

A way to defend landraces against genetic erosion and to conserve and utilize the genetic resources for the future is their conservation.

Two methods are used for the conservation of cereal genetic resources: ‘in situ’ and ex situ. Both conservation methods have benefits and limitations. The in situ conservation is carried out in “the natural habitats and include the maintenance and recovery of viable populations of species in their natural surroundings” on farms, while in the ex situ conservation, “the genetic resources are outside their natural habitats”, in identified gene banks or other national or regional conservation centres [21].

Ex situ germplasm collections could be used as base collections (by gene banks), and as working collections (by research/breeding institutions). The ex situ materials maintain the ‘genetic integrity’ of the accessions as they are not subjected to natural or farmers’ selection [22].

On the contrary, in situ is a dynamic conservation of cereal diversity in the centre of origin and in the areas in which that cereal species has been domesticated under the action of evolutionary forces (genetic drift, gene flow, mutations and selection), or simply traditionally grown, developing their distinctive properties and adaptation to environmental changes [17,23,24,25]. A consistent effort to create an inventory of in situ landrace conservation in Europe countries has been made in the frame of the Farmer’s Pride project (Horizon 2020 Programme of the European Union) [25]. The in situ approach supports two methodologies: (1) the genetic reserve, which is the natural long-term conservation sites of wild populations in their places of origin and (2) on-farm conservation, which is the sustainable management of genetic diversity (cultivars, wild and weedy species) in locally traditional agricultural systems [26,27]. The advantage of both in situ conservations, especially that on-farm, is to conserve wide variability in gene pools within species, providing a natural laboratory for the continued evolution of traits (i.e., adaptive response to climate change), and matching the economic benefits of farmers resulting from food traditions, local practices and social values [28,29,30].

For a comprehensive knowledge of cereal landrace diversity, it is essential to standardize the management strategies, starting from sampling procedures and guaranteeing a correct conservation of landrace diversities in gene banks over time. This way, the identification of agronomically valuable genes and untapped alleles to be used for breeding or pre-breeding purposes is facilitated [31,32].

The effectiveness of the ex situ conservation of landraces is closely linked to the possibility of sharing seeds and genetic information by a large community of users (e.g., gene bank curators, researchers, breeders, farmers and students) through a system based on their free and open access.

The European Cooperative Programme for Plant Genetic Resources (ECP-GR, http://www.ecpgr.cgiar.org/ (accessed on 30 March 2021)) is a good example of standardised procedures and compatible data documentation systems for better management, studies and exchange of genetic resources. Valuable collections of cereal landraces are held at several European and world gene banks.

The FAO website maintains the WIEWS on Plant Genetic Resources (PGR) (http://apps3.fao.org/wiews/wiews.jsp (accessed on 30 March 2021)), which contains metadata and data on germplasm collections and provides the identification and analysis of cereal landraces maintained in Catalogues around the world.

On a global scale, the GENESYS portal holds the information provided by three major international project partners: the European Cooperative Programme for Plant Genetic Resources (ECPGR-EURISCO), the System-wide Genetic Resources Programme (SGRP-SINGER) of the Consultative Group on International Agricultural Research (CGIAR) and the USDA Agricultural Research Service National Genetic Resources Program, accounting for approximately 2.4 million accessions held in ex situ collections worldwide (www.genesys-pgr.org (accessed on 30 March 2021)). In eastern Europe, one of the most important cereal collections is maintained at the Research Institute of Crop Production in the Czech Republic (http://genbank.vurv.cz/genetic/resources (accessed on 30 March 2021)).

The National Plant Germplasm System (NPGS) is a public germplasm collection that maintains seed samples, representing global diversity of small grains including wheat, barley, oat, rice, rye and triticale, and various wild relatives, including Aegilops. It is part of the Agricultural Research Service (ARS), the research agency of the United States Department of Agriculture (USDA) and is responsible for collecting, conserving, characterizing, evaluating, distributing and exchanging a rich and diverse genetic resources collection containing about 500,000 accessions. In particular, the wheat genetic resources are conserved at the National Small Grains Collection (NSGC), which is part of NPGS-ARS [33]. A partnership involving the Global Crop Diversity Trust, USDA, and Bioversity International, developed and deployed a gene bank documentation system called the Germplasm Resources Information Network (GRIN) (www.ars-grin.gov (accessed on 30 March 2021)). The GRIN database contains passport data, information which describes where and when an accession was collected, donated or developed and provides information on the availability and amount of seed that can be freely distributed to scientists and farmers in the US and around the world. Gene banks are therefore provided with a global management system that is powerful, flexible, easy to-use to safeguard plant genetic resources and to encourage their use by researchers, breeders and farmer-producers [33]. However, the typically small number of seeds that users can obtain from the GRIN system is only suitable for research use, and seeds need to be multiplied. Therefore, the continued production of landraces through on-farm conservation gives an enormous contribution in maintaining landraces diversity, ensuring timely availability of quality seed, and allowing for the dynamic evolution of landraces under diverse agro-ecosystems.

The main issue met by the gene bank users is the scarcity of information about some landrace characteristics, especially for some minor cereal species which play an important role in overall crop diversity [16]. Recent advances in evaluation and characterization of cereal landraces stored in gene banks, including molecular and biotechnological tools, offer new opportunities in the use of these genetic resources, thus avoiding redundant accessions [21]. Many other systems provide information on cereal crops: The International Barley Core Collection (IBCC); the International Maize and Wheat Improvement Centre (CIMMYT) for wheat and maize; the International Centre for Agricultural Research in the Dry Areas (ICARDA) for most cereals; the International Crop Information System (ICIS) that maintains data on wheat and barley; the Leibniz Institute of Plant Genetics and Crop Plant Research (IPK) for barley, oat and rye (https://web.archive.org/web/20120220095846/, http://www.icis.cgiar.org/icis/index.php/Main_Page (accessed on 2 April 2021)).

The CIMMYT germplasm bank contains over 170,000 wheat and 28,000 maize seed collections from across the world. These collections represent the genetic diversity of unique native varieties and wild relatives of maize and wheat and are studied and used as a source of currently untapped native diversity that could be used for plant improvement. The CIMMYT and the ICARDA wheat collections are the most important sources of landraces for wheat.

Recently, the Expert Working Group on Durum Wheat Genomics and Breeding, in the frame of the Wheat Initiative, and the International Durum Wheat Genome Sequencing Consortium developed two large germplasm collections: The Global Durum Wheat Panel (GDP) and the Tetraploid wheat Germplasm Collection (TGC), respectively [34,35]. Both collections are suitable for identifying beneficial alleles for traits of agronomic importance to be used in breeding and pre-breeding programs. The GDP panel contains 1028 accessions that mainly consists of *Triticum turgidum* ssp. *durum* modern varieties/advanced breeding lines, derived from worldwide breeding programs, durum wheat landraces (192), and a selection of wild and domesticated emmer wheats to maximize diversity. The TGC collection [34] consists of 1856 accessions designed to cover most of the variation present in tetraploid wheats, based on the analysis of accession passports, and comprises wild and domesticated emmer, durum wheat landraces and cultivars, and tetraploid subspecies. Small seed amounts of the GDP, TGC and of a Tetraploid Core Collection (TCC), which comprises 432 accessions capturing over 95% of the GDP and TGC biodiversity, is available for research, breeding and training purposes at the developers, under standard MTA terms and conditions. Furthermore, genotypic data related to thousands of single-nucleotide polymorphism (SNP) markers [36] are also freely available at the GrainGenes site (https://wheat.pw.usda.gov/GG3/global_durum_genomic_resources (accessed on 2 April 2021)).

## 3. Exploration of Genetic Diversity and Population Structure

Selection activities in the frame of the ongoing breeding programs led to a reduction of the diversity in genetic materials and are considered as a bottleneck in crop evolution after the domestication process. This assumption is accompanied by the idea that many alleles useful for breeding could have been lost in the selection process. Therefore, in the last few years a growing interest has focused on analysing genetic diversity in landrace collections in cereal crops.

In some cases, these studies were aimed at characterizing a limited number of important genotypes, such as those traditionally grown by farmers in particular areas. The genetic analyses, carried out on a proper number of individuals for each accession, often showed a certain degree of heterogeneity in each landrace. As an example, Mangini et al. [37] carried out a phenotypic and molecular analysis of three durum and one common wheat Italian landrace population, and the SNP characterization revealed different haplotypes within each landrace, indicating a genetic structure based on a mixture of genotypes. In other cases, landraces were maintained as inbred lines, and analysis on very large collections became possible. These analyses were often focused on panels of landraces with a specific geographical origin, as in the case of durum or common wheat landraces from Ethiopia [38,39], Sicily [40], Morocco [41], Iran [42], Palestine and Israel [43], Pakistan [44], Turkey [45] and Mexico [46]; barley from Nepal [47], the Canary Islands [48], Tunisia [49,50] and Jordan [51]; oat from Poland [52] and Spain [53]; rice from Pakistan [54] and India [55] and millets from Senegal [56] and China [57,58]. If focusing on specific geographic areas has the advantage of exploring within a range of genotypes well adapted to that environment, examining wider collections opens the possibility of investigating the genetic relationship across landraces spread around the world, and having a more precise estimation of the genetic diversity within the group of landraces and with respect to advanced breeding lines or modern cultivars. To quote some examples, studies carried out on panels of hundreds of landraces have been considered in durum and common wheat [35,59,60], barley [61,62] and rye [63]. In general, a higher genetic diversity has been observed in the group of landraces compared to the groups of advanced breeding lines and modern cultivars, indicating landraces as a useful source of variation for breeding. Additionally, when clustering and population structure analyses have been considered, the total genetic variation was higher within than between groups, and the groups were in general consistent with the geographical origin of the lines, except in a few cases. Mzid et al. [64] assessed genetic diversity in a panel of 53 Lebanese barley landraces through the electrophoretic pattern of the seed storage proteins, hordeins. In this case, the absence of correlation between the genetic variability and the geographic origin of sample provenance was explained by the fact that Lebanon is a small country where seeds are easily exchanged between farmers’ communities in the different regions. Similarly, Yadav et al. [47] phenotypically evaluated 25 naked barley landraces from different regions of Nepal. The UPGMA cluster analysis, carried out with qualitative phenotypic descriptors and quantitative traits, categorized the landraces in five clusters with no distinct regional grouping patterns. In this case, principal component analysis revealed the quantitative traits, such as grain yield, plant height and earliness, and qualitative traits, such as grain colour, lemma awn/hood and lemma awn barbs, to be the principal discriminatory characteristics of the Nepalese naked barley landrace collection.

Phenotypic evaluation of landraces is important to identify sources of useful loci for traits of interest in breeding and pre-breeding programs, in relation to traits with a simple genetic basis as the resistance to diseases, but also to complex traits such as grain yield (reviewed by Dwiwedi et al. [5]). Nevertheless, a clearer picture, in terms of genetic diversity, can be achieved using molecular markers. Markers based on polymorphisms at the level of seed storage proteins have been used in different cereal species such as Ethiopian emmer accessions [49], durum wheat and barley landraces [64,65]. Molecular markers based on DNA have been developed, both at the chromosome and the DNA sequence level. Polymorphisms were identified at the level of chromosome banding, through cytofluorometry [66,67]. The analysis of 58 varieties and landraces demonstrated a remarkable reproducibility and sensitivity of flow cytometry for the detection of numerical and structural chromosome changes [68]. In this regard, the dissection of complex genomes by flow cytometric sorting into the individual chromosomes reduces its complexity in a lossless manner, having a significant impact in many areas of research and giving a strong impulse to the sequencing of complex plant genomes [69,70,71]. At sequence level, DNA-based molecular markers have become the most suitable tool in this kind of study, thanks to their informativeness and to the great reduction in time processing and costs observed in the last few years. Random Amplified Polymorphic DNA (RAPD) markers were initially used for assessing genetic diversity in cereal landraces [49], but they were characterized by a low reproducibility, therefore Simple Sequence Repeat (SSR) markers became the method of choice thanks to their reproducibility and informativeness with a high number of alleles detected per locus. As an example, 8.1 alleles per locus were detected in a panel of 66 barley landraces from Tunisia [50], and 14.6 alleles per locus were identified in a collection including 36 oat commercial varieties and 141 landraces from Spain [53]. In more recent times, high-throughput methods have been developed, such as those based on fixed markers arrays, which include Diversity Array Technology (DArT) markers and SNP arrays. These methods have been shown to be suitable for genetic studies on cereal landraces and can assess a large number of entries, as in the case of panels with several hundreds of durum wheat landraces from Spain, assessed with DArT markers, or from Ethiopia and different countries worldwide, tested with SNP arrays [35,39]. An important aspect is that a certain ascertainment bias should be considered, as these platforms were mainly developed starting from cultivars [34]. For this reason, methods based on Genotyping by sequencing, including DArTseq, are also used in this kind of study [46,72]. The use of high-throughput markers allowed, in particular, the collection of more precise information on decay of linkage disequilibrium in landrace panels, which showed a higher resolution compared to commercial cultivars, for their use in association mapping analysis [51,73]. Moreover, the availability of a large number of markers with a good coverage of the genome is important to identify rare and private alleles, which are present only in a defined group of genotypes [35,36,37,38,39,40,41,42,43,44,45,46,47,48,49,50,51,52,53,54,55,56,57,58,59,60]. This kind of knowledge is very important for breeding, as landraces can be chosen not only based on their diversity per se, but also for specific alleles of interest in a particular breeding program.

## 4. Landraces as a Genetic Resource to Identify the Genetic Factors Responsible for Resistance to Biotic and Abiotic Stresses

The evolution of plant breeding with the consequent genetic erosion and the gradual shift towards a model of agriculture based on genetic uniformity results in the need to re-gain genetic variability to adapt crops to climate changes [74]. The importance of keeping diversity in breeding programmes has been well established. The possibility of accessing the information present in the gene banks offers a significant contribution to the identification of genes/alleles useful in the populations of landraces preserved in the various ex situ collections.

### 4.1. Abiotic Stresses and Traits of Agronomic Importance in Limiting Environments

The identification of abiotic stress tolerant alleles in landraces of cereal crops through mapping and GWAS approaches is of great importance to improve cereal crop adaptation to stress-prone environments. Two types of studies have been carried out in this regard: on one hand, traits directly associated to tolerance to abiotic stresses have been analysed by assuming their importance in improving the agronomic performance of crops in stress-prone environments. On the other hand, landraces have been evaluated for grain yield and quality, or related traits in limited environments. For the first kind of investigation, in rice, the discovery of submergence-tolerant landrace ‘FR13A’ led to the identification of the locus *SUBMERGENCE 1* (*SUB1*) located on chromosome 9, which codes for ethylene response factor [75]. The positional cloning of *SUB1* locus revealed three genes: *SUB1A*, found in tolerant lines, and *SUB1B* and *SUB1C*, found only in intolerant lines [76,77]. In turn, it was found that *SUB1A* has two allelic forms, *SUB1A-1*, associated with tolerant lines, and *SUB1A-2*, associated with intolerant line. Two QTLs for drought tolerance, based on leaf wilting, were recently identified on chromosomes 2H and 5H in the Chinese barley landrace ‘TX9425’ [78], which account for 42% and 14% of phenotypic variation, respectively. The QTL on 2H was closely linked with a gene controlling ear emergency, while the candidate gene underlying the QTL on 5H was suggested to be *9-cis-epoxycarotenoid dioxygenase 2* (*HvNCED2*), which is involved in the synthesis of abscisic acid. In another study of GWAS, two candidate genes, *HvCBF10B* and *HvCBF10A*, underlying this QTL were identified, which have regulatory function under drought condition [79]. Attempts to apply GWAS to drought resistance are limited due to the intrinsic complexities of investigating drought stress and its associated responses. Using 645 wheat landraces collected from 10 Chinese agroecological zones, Lin et al. [80] identified 26 QTLs associated with drought through the evaluation of 16 seedling traits related to root and shoot growth and water content under normal and drought (induced by polyethylene glycol) conditions. Extremely resistant and sensitive accessions were identified for future drought resistance breeding and further genetic analyses.

Rice productivity in both rain-fed and irrigated agro-ecosystems is also affected by salt stress. Rice landraces ‘Nona Bokra’ and ‘Pokkali’ are excellent sources of salt tolerance. Nona Bokra contributed a major QTL for shoot K^+^ concentration on chromosome 1 (SKC-1) [81], and additive QTLs with small effects, mainly affecting Na^+^/K^+^ ratio [82,83]. The SKC-1 gene, isolated by map-based cloning, encodes a sodium transporter that control K^+^/Na^+^ homeostasis under salt stress [81]. Pokkali contributed a major QTL, *Saltol1*, associated with Na^+^/K^+^ ratio and salinity tolerance [84] and additive QTLs associated with Na^+^ and K^+^ concentration and with salt injury score [85]. Further researches revealed that Saltol1 is a complex locus, mapped on chromosome 1, with multiple Pokkali alleles regulating shoot Na^+^/K^+^ homeostasis [86,87]. Similarly, the barley landrace ‘TX9425’ contributed a major QTL for salinity tolerance on chromosome 7H, explaining 28% of phenotypic variation estimated by plant survival under salt stress [78], and a significant QTL on chromosome 2H that explains 45% of phenotypic variation in the potting mixture trials, using plant survival and leaf chlorosis as evaluation criteria [88]. Finally, another salt tolerant locus, *HvNax4*, was identified on chromosome 1HL in the Algerian landrace ‘Sahara 3771’ [89].

Another trait, potentially limiting crop production, is boron toxicity. Tolerance to toxicity is associated with the ability to maintain low boron concentrations in the shoot [90]. The *Bot1* gene, responsible for the high boron-toxicity tolerance of the Algerian barley landrace ‘Sahara 3771’, was identified [91]. In bread wheat, the boron tolerant landrace ‘G61450’ contributed the boron toxicity gene, *Bo4*, which was mapped on chromosome 4AL [92].

Cereal landraces are also important sources of beneficial alleles for grain yield and quality in low-producing environments. For this reason, collections of landraces have been assessed in mapping studies to identify genetic determinants for these traits. As grain yield is a trait with a very complex genetic basis and a strong genotype x environment interaction, in some cases traits which are strongly correlated with yield have been considered. As an example, different leaf traits were assessed in a panel of 180 Vietnamese rice landraces in controlled conditions, such as leaf dry matter percentage, which can be considered a proxy for the photosynthetic efficiency per unit leaf area, contributing to yield [93]. Genetic analysis with more than 21,000 SNP markers led to identified QTLs, some of which were in a position where genes with a known function in leaf development or physiology were located. Similarly, Ta et al. [94] analysed several traits related to panicle architecture, one of the key components of rice yield, in a panel of Vietnamese landraces.

Numerous studies have focused on the evaluation of grain yield and yield components directly. Huang et al. [95] identified ~3.6 million SNPs by sequencing 517 rice landraces and performed GWAS for 14 agronomic traits based on a high-density haplotype map of the rice genome. Many chromosomal regions were mapped in this study, as the overall genetic variation observed in this panel represented at least 80% of the world’s rice cultivars. In this case, characterizing a large panel of cereal landraces with a high-density marker system, based on genome re-sequencing, provided useful information not only on genetic determinants of traits of agronomic importance, but also on genetic relationships across groups of genotypes adapted to various agro-climatic conditions. A panel of 150 Jordanian landraces was evaluated for yield and yield components in Jordan under rain-fed conditions [51]. The GWAS analysis allowed the identification of three significant QTLs located at 1H, 2H and 7H, important for grain yield in dry environments. Moreover, three accessions with high yield and stability across environments were identified [51]. Studies in which favourable and limiting environments were compared allowed the identification of genomic regions specifically involved in sustaining grain yield and quality in difficult conditions. Alleles that were adaptive under drought stress conditions for a number of agronomic traits, including yield, were identified in a collection of 298 Iranian bread wheat varieties and landraces [96] (Rahimi et al. 2019). Fourteen large-effect QTLs for grain yield associated with drought adaptation were identified in rice landraces, six of which were effective in multiple genetic backgrounds and environments [97]. A set of 472 rice genotypes comprising landraces and breeding lines was evaluated under field conditions with low and recommended nitrogen to identify genotypes with relative higher yield under low nitrogen, together with 12 genomic regions for yield and yield associated traits and three candidate genes from QTL regions showing enhanced expression in the genotypes with promising yield under low N [98]. As regards phosphorus deficiency, widespread in tropical soils, the well-known gene *Phosphate uptake 1* (*Pup1*), identified in the rice landrace ‘Kasalath’ and located on chromosome 12, increases phosphorus uptake and confers significant grain yield advantage in phosphorus deficient soils [99,100]. *Pup1* is found in landraces or cultivars adapted to drought-prone environments [101] and it is effective in different genetic backgrounds and environments [102]. A study on the functional mechanism of *Pup1* revealed the presence of a Pup1-specific protein kinase gene, named *Phosphorus starvation tolerance 1* (*PSTOL1*), which is absent in intolerant cultivars. The overexpression of *POSTL1* significantly enhances grain yield in phosphorus deficient soils, promoting early root growth, thereby enabling plants to acquire more phosphorus and other nutrients [103].

For a good agronomic performance in stress-prone environments, it is important to sustain not only grain yield but also quality. A good variation has been found in landraces as an example for storage proteins in wheat grain [104]. In the last few years in particular, a great interest has arisen for traits related to the nutritional quality of cereal grain for human nutrition. A core set of 190 rice landraces was used to decipher the genetic structure and to discover the chromosomal regions containing QTLs affecting the grain micro-nutrients and fatty acids, as well as yield-related traits [105]. A total of 22 significant QTLs were identified, comprising those involved in the control of content of Zn, oleic acid and Fe. Landraces with a strong expression of the traits analysed in this study and the closely linked molecular markers represent a valid tool for the use of these QTLs in rice breeding for developing new varieties with high yield and nutritional value.

These results confirm that landraces, thanks to their long evolutionary history and adaptation to stressful environments, are ideal genetic resources to explore novel genetic variation for responses to environmental constraints. In particular, landraces are an effective source of useful alleles to sustain grain yield and quality in both favourable and limiting environments. In some cases, the loci involved in the control of yield in good conditions can still maintain a good level of production when stresses are mild [106]. In other cases, alleles which specifically express in environments with more pronounced stress conditions have been identified in landraces, which can help in breeding for improved lines well adapted to specific areas.

### 4.2. Biotic Stresses

Plant diseases are serious constraints to the production of cereal crops. During the vegetation period, the largest infections are caused by pathogenic fungi. Powdery mildew and rusts of cereals and grasses are the most dangerous diseases of wheat and barley. Head blight caused by different fungi of the genus Fusarium also affect wheat, rye, triticale, barley and oat crops, but also maize. Genetic improvement of resistance to pathogens through breeding represents the best economical and eco-friendly alternative to minimize yield losses. Many studies aimed at identifying resistance genes/loci against various diseases are available for cereals. Most of these studies were based on the analysis of biparental populations and, more recently, GWAS has been employed. Landraces may carry new sources of resistance that can be exploited to enrich the narrow resistance spectrum currently found in adapted cultivars. Studies that have reported screening with molecular markers linked to specific resistance genes of panels including landraces grown in a particular geographic area are available, such as the collection of rice landraces and breeding lines from India evaluated for 22 genes against the fungus *Magnaporthe oryzae*, from which two landraces emerged for high resistance to blast, and were therefore useful in breeding programs [107]. In particular, the landrace Tetep was the donor of the *Pi54* gene for broad-spectrum blast resistance, which has been cloned, and transgenic lines harboring *Pi54* showed a high degree of resistance to diverse strains of blast pathogen [108].

Many studies that have focused on landraces as a good reservoir of resistance genes against rusts are available for wheat [109,110,111,112,113,114,115,116,117,118,119,120,121]. The Portuguese durum wheat landrace PI 192051 has been used to map leaf and stem rust resistance QTLs on chromosomes 4A and 7A, respectively, and to develop SNP markers tightly linked to the identified loci [122]. A GWAS was performed using 152 wheat landraces from China to identify effective stripe rust resistance loci, which resulted in 19 accessions displaying stable and high degrees of resistance to stripe rust development when exposed to mixed races of Pst at the adult-plant stage in multi-environment field assessments, and 40 QTL regions for adult-plant resistance [123]. A multi-pathogen resistance gene, *Lr67*, which confers partial resistance to all three wheat rust pathogen species (*Pt*, *Pst*, *Pgt*) and powdery mildew (*Bgt*), as demonstrated by using a combination of comparative genomics, mutagenesis and transformation studies, was isolated from a bread wheat landrace (PI250413) [124]. Wheat landraces have also been studied for other diseases, including Fusarium Head Blight [125], barley yellow dwarf (BYD) [126], powdery mildew [127,128,129] and stem sawfly [130]. A recombinant inbred line (RIL) population derived from Haiyanzhong, a Chinese wheat landrace showing a high level of resistance to FHB spread within a spike (type II resistance), has been used to map six QTLs (one major and five minor) and obtain KASP markers useful for MAS [125]. In particular, it is known that Germplasm from East Asia harbours highly resistant genotypes, including landraces (e.g., Wangshuibai, Nobeokabozukomugi) [131]. Indeed, Wangshuibai is an FHB-resistant Chinese landrace unrelated to cv. Sumai 3, the most commonly used FHB-resistant source, and it was the source of two major type I resistance FHB resistance QTLs, named *Fhb4* and *Fhb5*, that were fine mapped by using NIL populations [132,133].

Landraces from China were also considered very good sources of powdery mildew resistance. For example, the Chinese wheat landrace Xuxusanyuehuang has been found by comparative genomics analysis to possess a single recessive powdery mildew resistance gene, *Pm61*, on chromosome 4A [128], whereas the landrace Duanganmang has been used to map a new gene, *PmDGM*, conferring powdery mildew resistance [134]. Resistance genes *Pm24*, *Pm24b* and *MlHLT* were identified in wheat landraces Chiyacao, Baihulu and Hulutou, respectively [135]. In particular *Pm24* was map-based cloned, and it was found to be a rare natural allele of tandem kinase protein (TKP) with putative kinase pseudokinase domains. A 6-bp deletion at the kinase domain was considered critical for the gain of powdery mildew disease resistance [135]. Finally, the gene *Pm3b*, originating from the hexaploid wheat landrace Chul, was found by positional cloning to be a member of the coiled-coil nucleotide binding site leucine-rich repeat (NBS-LRR) type of disease resistance genes [136]. Regarding barley landraces, most studies are focused on resistance against fungus *Blumeria graminis* f. sp. *Hordei* [137,138,139]. The *mlo* (Mildew resistance locus o)-based resistance is considered the most reliable weapon to protect plants from infection by this fungus [140]. Loss of function of one or more of such genes is associated with plant immunity. Ethiopian landraces of barley were the first known examples of natural *mlo* mutants [140]. Moreover, three QTLs conferring broad spectrum resistance to powdery mildew were identified on chromosomes 7HS, 7HL and 6HL in the Spanish barley landrace-derived lines SBCC097 and SBCC145 [137], whereas the barley line 2553-3, selected from a Moroccan landrace, has been reported to possess a new resistance gene, named *MlMor* [139]. QTL/genes for net blotch disease [141,142], stem rust [143], barley yellow mosaic virus (BaYMV), barley mild mosaic virus (BaMMV) [144] and barley scald [141] have also been documented.

Very recently, a genetic analysis of a worldwide barley collection, including 277 landraces, for resistance to net blotch disease (*Pyrenophora teres* f. *teres*) has been carried out, resulting in 15 QTL regions, four of which had never been described in previous studies [142]. Finally, stem rust resistance has been characterized in barley landraces, in particular against the African TTKSK race, and the *rpg4/Rpg5* locus has been indicated to be involved in conferring resistance [143].

Few genetic studies for disease resistance in maize and oat landraces are available. The well-known gene *Htn1*, reported to code a wall-associated receptor-like kinase by high-resolution map-based cloning, represents an important source of genetic resistance against northern corn leaf blight that was originally introduced from a Mexican landrace into modern maize breeding lines in the 1970s [145]. Very recently, two European maize landraces were analysed individually for *Gibberella* ear rot (GER) resistance using genome-wide association studies and genomic selection (GS) [73]. Loci with small effects were found, and for two SNPs candidate genes were proposed belonging to functional groups, including binding activity, kinase activity, response to stress/stimulation, signal transduction, catalytic activity and metabolic and biosynthetic processes. Moreover, two RIL populations were constructed to elucidate the genetic basis of resistance to Maize rough dwarf disease (MRDD), a significant viral disease caused by rice black streaked dwarf virus (RBSDV), resulting in the resistance QTL (qZD-MRDD8-1) with the largest effect (more than 23% of the phenotypic variability observed) [146]. Finally, Montilla-Bascòn et al. [147] analysed, by GWAS, a panel of 177 oat accessions, including cultivars and landraces, for crown rust and powdery mildew, providing markers as good candidates for MAS.

In conclusion, cereal landraces have a great potential as sources of novel disease resistance genes, and a good combination of these genes could help to alleviate diseases. Therefore, more efforts are needed to utilize genomic approaches in order to exploit genetic variability across landrace collections worldwide.

The QTLs/Genes for abiotic and biotic stresses identified in cereal landraces have been summarized in Table 1.

## 5. Use of Landraces in Cereal Breeding

Plant breeders who want to create new high-yielding varieties tend to make crosses among élite lines where they have the highest likelihood of developing new varieties. Currently, climate change is affecting grain yields worldwide, threatening food quality and security. The cereals grown in many areas worldwide, such as the Mediterranean area, are often exposed to biotic and abiotic stresses, such as high temperatures and water stress, especially during the filling kernels period. Compared to modern cultivars, landraces are often resilient to stresses and represent a valuable source of germplasm for meeting the future needs of sustainable agriculture in the context of climate change [16,148,149].

**Figure 1:**
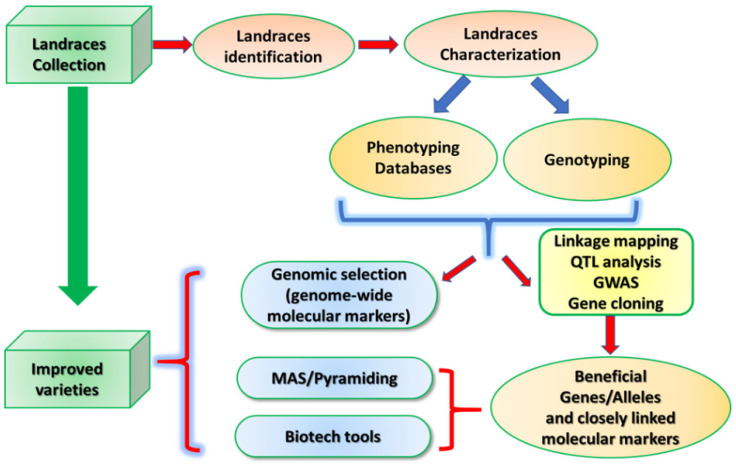
Schematic workflow to get improved varieties starting from landraces.

The wide genetic variability and high potential for adaptation to extreme climatic conditions of landraces make them important for the genetic improvement of cereals, particularly in improving agronomic traits such as yield and yield stability [150,151,152,153]. Indeed, they can provide new genes or ‘new’ allelic variants for known functional genes which could be introgressed into modern varieties by hybridization and other approaches in breeding programs [153]. For these reasons, it is important to monitor the genetic diversity maintained in the landraces, intensifying phenotypic and genetic characterization to find new beneficial alleles/loci for the traits of interest to use in breeding programmes [154] (Figure 1).

Improving resistance to biotic stresses is, in general, a simple task, as many resistance genes provide a complete, even if race-specific, resistance. Examples are available in different cereal species. *Yr51*, associated to marker *sun104* in wheat landrace AUS27858, is currently backcrossed into Australian and Indian wheat cultivars through marker-assisted selection, to improve resistance to yellow rust [109]. As single gene-based resistance can be easily overcome by new virulent pathogen races; the pyramiding of several R genes into the same genetic background has been recognized as an efficient strategy to increase and prolong disease resistance in cultivars. As many resistance genes have been mapped in the landraces of different crops, they can be used as valuable sources in pyramiding breeding programs. Interestingly, in recent years, single genes providing durable and multi-pathogens resistance have been identified and cloned. One example is the landrace-derived *Lr67* gene, which was introduced into wheat cultivar Thatcher to produce the near-isogenic Thatcher + Lr67 line, RL6077, confirming a resistance phenotype to a number of races of wheat rusts and powdery mildew [124,155]. The availability of genome sequences of different species has made possible the development of functional markers to be used in breeding. For example, for one of the major blast resistance genes in rice, *Pi54*, a PCR-based co-dominant molecular marker targeting an InDel identified in the exonic region of the gene, co-segregating with blast resistance, has been developed and used for routine deployment in MAS in breeding programs [156,157].

Improving tolerance to abiotic stresses is a more complex issue due to the quantitative nature of this trait. Nonetheless, landraces have also been shown to be valuable sources of beneficial alleles in this case.

Extensive studies have been done in rice, given the importance of this cereal in world food supply. *SUB1A-1* allele, found only in flooding tolerant lines, has been introgressed through MAS into locally adapted and widely grown rice cultivars in Asia [158], improving grain yield with no yield penalty under non-flooding [159]. This locus has also been pyramided with the *Saltol1* allele, associated with Na/K ratio to obtain rice cultivars highly tolerant to salt stress [160]. Good results have also been obtained for tolerance to drought stress. Indeed, the pyramiding of large-effect QTLs associated to drought tolerance and carried by landraces led to the development and release of 17 height-yielding drought-tolerant rice cultivars in Asia and Africa [97]. Other environmental constraints are due to deficiency of nutrients or the presence of toxic metals in the soil. Rice has been greatly improved for phosphorus uptake through the introgression into elite cultivars of *Posphate uptake 1* (*Pup1*) from the rice landrace Kasalath [100]. These lines showed significantly increased grain yield on P-deficient soils [102]. In barley, the locus *Bot1* responsible for the high boron-toxicity tolerance was introgressed by MAS into commercial barley cultivars, and the lines obtained showed lower yield than the recipient cultivars, which have been further tailored to develop lines carrying recombination events directly adjacent to *Bot1* for use in barley breeding [91]. Improving grain yield is a very complex task due to the high number of yield components and loci involved in their genetic control. Usually, landraces are a valuable source for beneficial alleles to improve yield in low-producing environments. Monteagudo et al. [161] improved an élite cultivar of barley through the introgression of alleles from two local landraces, SBCC042 and SBCC073, selected from the Spanish Barley Core Collection (SBCC), with high performance under low productivity conditions. Favourable alleles from both landraces have been mapped on chromosome 1H, encompassing the *HvFT3* gene, which contributed to an acceleration of flowering and an increase in thousand-kernel weight (TKW), and on chromosome 6H, which contributed to increasing plant height and TKW. Favourable alleles responsible for an increased ground coverage after winter, which could be exploited as an adaptive trait, were contributed only by SBCC042. South American maize landraces are known for high-altitude adaptation and tolerance to abiotic stresses [162]. Recently, the introgression of alleles from different landraces into adapted maize cultivars has led to building new populations that are more productive than recurrent parents, which can be used to develop cultivars and parental lines of hybrids combining drought adaptation and high grain yield [162,163,164]. In general, breeding programs aim to increase crop yield while maintaining, as much as possible, the grain quality features of the lines. A successful example in this regard is the obtainment of improved lines from the sorghum landrace M35-1, which out-yielded its parent in the Indian drought-prone environment while maintaining its grain quality [165]. Nonetheless, landraces can also contribute to actively improving grain quality. A classic example is the *Opaque-2* mutation, which improves kernel protein quality, and was identified in a Peruvian maize landrace [166].

Despite the scientific literature on the role of cereal landraces in breeding, landrace potential has not been fully exploited in modern breeding [167], as indicated by a study on a worldwide wheat landrace collection, analysed with high throughput genotyping platforms, that revealed a substantial amount of novel genetic diversity in the landraces, which is either not captured in current breeding programs or has been lost due to previous selection pressures [168]. The reduced utilization of durum landraces in breeding programs has also been confirmed by Lodhi et al. [169]. Given the complexity of the problem, it is clear that new strategies are required to overcome the limits of traditional approaches, such as those based on the development of introgression lines, which is slow and affected by the loss of genetic variability occurring when a new population is established by a very small number of individuals from a larger population, or the negative effect of epistasis when dealing with complex multigenic traits.

## 6. Conclusions

The use of cereal landraces as a genetic resource for breeding is a response to some negative consequences of domestication, modern agricultural practices and conventional farming, which led to the loss of genetic diversity and a reduction of yield in unfavourable environments. 

Most of the genetic diversity present in cereal landraces is still little known and even less used. A major part of this valuable landrace diversity is conserved in the farmers and the world’s gene bank networks and should be largely exploited for traits of interest. The information derived from gene banks, phenotypic assessment and high-throughput genetic tools is valuable for the choice of parental lines to use in crosses aimed at obtaining improved varieties, especially for enhancing the adaptation and productivity of crops in situations of global climate change, to increase the sustainability of cereal crop production in the future.

## Figures and Tables

**Table 1:** QTLs/Genes for abiotic and biotic stresses identified in cereal landraces.

Species	Landrace (Origin)	Gene/QTL	Trait	Analysis Type	Refs.
**Abiotic Stress**					
Wheat	G61450 (Greece)	*Bo4*	Boron tolerance	Molecular mapping	[92]
Rice	FR13A (India)	*SUB1*	Submergence tolerance	Molecular mapping	[75]
Rice	Aus, Indica, and Basmati accessions (India)	14 QTLs	Drought tolerance	Molecular mapping	[97]
Rice	Nona Bokra (India)	*qSKC-1*	Salinity tolerance	Molecular mapping	[81]
Rice	Nona Bokra (India)	16 QTLs	Salinity tolerance	Molecular mapping	[82,83]
Rice	Pokkali (India)	*Saltol1*	Salinity tolerance	Molecular mapping	[84]
Rice	Pokkali (India)	10 QTLs	Salinity tolerance	Molecular mapping	[85]
Rice	Kasalath (india)	*PUP1*	Phosphorus uptake increase	Molecular mapping	[99,100]
Barley	TX9425 (China)	2 QTLs	Drought tolerance	Molecular mapping	[78]
Barley	Accessions from diverse geographic areas	*QRdw.5H*	Drought tolerance	GWAS	[79]
Barley	Chinese accessions	26 QTLs	Drought tolerance	GWAS	[80]
Barley	TX9425 (China)	1 QTL	Salinity tolerance	Molecular mapping	[78]
Barley	TX9425 (China)	1 QTL	Salinity and waterlogging tolerance	Molecular mapping	[88]
Barley	Sahara 3771 (Algeria)	*HvNax4*	Salinity tolerance	Molecular mapping	[89]
Barley	Sahara 3771 (Algeria)	*Bot1*	Boron tolerance	Molecular mapping	[91]
**Biotic Stress**					
Wheat	AUS27858 (Australia)	*Yr51*	Stripe rust resistance	Molecular mapping	[109]
Wheat	CItr 4311 (Iran)	*Sr9h*	Stem rust resistance	Molecular mapping	[110]
Tetraploid Wheat	Accessions from diverse geographic areas	35 QTLs	Stem rust resistance	GWAS	[111]
Wheat	Ethiopian accessions	24 QTLs	Stem and stripe resistance	GWAS	[113]
Wheat	PI 182103 (Pakistan)	*Yr79*	Stripe rust resistance	Molecular mapping	[114]
Wheat	Americano 44 (Uruguay)	3 major QTLs	Leaf rust resistance	Molecular mapping	[115]
Tetraploid Wheat	Accessions from diverse geographic areas	22 QTLs	Stem rust resistance	GWAS	[116]
Wheat	PI 362698 (Montenegro)	5 QTLs	Stem rust resistance	Molecular mapping	[117]
Wheat	Pingyuan 50/Mingxian 169 (China)	8 QTLs	Leaf rust resistance	Molecular mapping	[118]
Wheat	Iranian accessions	54 QTLs	Stem rust resistance	GWAS	[119]
Wheat	ICARDA accessions	19 QTLs	Stripe rust resistance	GWAS	[120]
Wheat	Mexican accessions	17 QTLs	Stripe and stem rust resistance	GWAS	[121]
Durum Wheat	PI 192051 (Portugal)	2 major and 3 minor QTLs	Leaf and stem rusts resistance	Molecular mapping	[122]
Wheat	Chinese accessions	40 QTLs	Yellow rust resistance	GWAS	[123]
Wheat	PI 250413 (Pakistan)	*Lr67*	Leaf, stem and stripe rust, and powdery mildew resistance	Mutagenesis and transformation	[124]
Wheat	Haiyanzhong (China)	6 QTLs	Fusarium Head Blight resistance	Molecular mapping	[125]
Wheat	Chinese and Australian accessions	36 QTLs	Barley yellow dwarf virus resistance	GWAS	[126]
Wheat	Hulutou (China)	MlHLT	Powdery mildew resistance	Molecular mapping	[127]
Wheat	Xuxusanyuehuang (China)	*Pm61*	Powdery mildew Resistance	Molecular mapping	[128]
Wheat	Honghuaxiaomai (China)	*PmHHXM*	Powdery mildew resistance	Molecular mapping	[129]
Wheat	Duanganmang (China)	*PmDGM*	Powdery mildew resistance	Molecular mapping	[134]
Wheat	Chiyacao, Baihulu Hulutou (China)	*Pm24*, *Pm24b*, *MIHLT*	Powdery mildew resistance	Molecular mapping	[135]
Wheat	ChuI	*Pm3b*	Powdery mildew resistance	Positional cloning	[136]
Durum Wheat	PI 166471 (Turkey)	9 QTLs	Stem sawfy resistance	Molecular mapping	[130]
Wheat	Wangshuibai (China)	2 QTLs (*Fhb4*, *Fhb5*)	Fusarium Head Blight resistance	Molecular mapping	[132,133]
Rice	Tetep (Vietnma)	Pi54	Blast resistance	Molecular mapping	[108]
Barley	SBCC097/SBCC145 (Spain)	3 QTLs	Powdery mildew resistance	Molecular mapping and positional cloning	[137]
Barley	Chevallier (UK)	5 QTLs	Powdery mildew resistance	Molecular mapping	[138]
Barley	Landrace 255 (ICARDA No. ICB 31956) (Morocco)	*MlMor*	Powdery mildew resistance	Molecular mapping	[139]
Barley	Ethiopian and American accessions	51 QTLs	Leaf scald and net blotch	GWAS	[141]
Barley	Accessions from diverse geographic areas	15 QTLs	Net blotch	GWAS	[142]
Barley	Hv501, Hv545, Hv602, Hv612 (Switzerland)	*Rpg5*	Stem rust	BSA	[143]
Barley	HOR3298 (Iran)	*eIF4E*	Barley yellow mosaic virus	BSR-seq	[144]
Maize	Kemater Landmais Gelb/Petkuser Ferdinand Rot (Germany)	8 QTLs	Gibberella ear rot	GWAS	[73]
Maize	Pepitilla (Mexico)	*Htn1*	Northern Corn leaf blight	Map-based cloning	[145]
Maize	D863F/ZS301 (China)	10 QTLs	Rough dwarf disease	Molecular mapping	[146]
Oat	Spanish accessions	6 QTLs	Crown rust and powdery mildew	GWAS	[147]

GWAS = Genome-wide Association study; BSA = Bulked segregant analysis; BSR = Bulked segregant RNA.

## Data Availability

No new data were created or analyzed in this study. Data sharing is not applicable to this article.
